# Mapping and functional characterization of the murine Smoothelin-like 1 promoter

**DOI:** 10.1186/1471-2199-12-10

**Published:** 2011-02-27

**Authors:** Annegret Ulke-Lemée, Sara R Turner, Saad H Mughal, Meredith A Borman, Robert J Winkfein, Justin A MacDonald

**Affiliations:** 1 Smooth Muscle Research Group and Department of Biochemistry & Molecular Biology, University of Calgary, 3280 Hospital Drive NW, Calgary, Alberta, T2N 4Z6, Canada

## Abstract

**Background:**

Smoothelin-like 1 (SMTNL1, also known as CHASM) plays a role in promoting relaxation as well as adaptive responses to exercise, pregnancy and sexual development in smooth and skeletal muscle. Investigations of *Smtnl1 *transcriptional regulation are still lacking. Thus, in this study, we identify and characterize key regulatory elements of the mouse *Smtnl1 *gene.

**Results:**

We mapped the key regulatory elements of the *Smtnl1 *promoter region: the transcriptional start site (TSS) lays -44 bp from the translational start codon and a TATA-box motif at -75 bp was conserved amongst all mammalian *Smtnl1 *promoters investigated. The *Smtnl1 *proximal promoter enhances expression up to 8-fold in smooth muscle cells and a second activating region lays 500 bp further upstream. Two repressing motifs were present (-118 to -218 bp and -1637 to -1869 bp). The proximal promoter is highly conserved in mammals and contains a mirror repeat sequence. *In silico *analysis suggests many transcription factors (notably MyoD) could potentially bind within the *Smtnl1 *proximal promoter sequence.

**Conclusion:**

*Smtnl1 *transcript was identified in all smooth muscle tissues examined to date, albeit at much lower levels than found in skeletal muscle. It is unlikely that multiple SMTNL1 isoforms exist since a single *Smtnl1 *transcription start site was identified in both skeletal and intestinal smooth muscle. Promoter studies suggest restrictive control of *Smtnl1 *expression in non-muscle cells.

## Background

The smoothelin-like 1 (SMTNL1 [Swiss-Prot: Q99LM3]) protein was discovered as a novel protein phosphorylated in response to cGMP stimulation of ileal smooth muscle tissue [1]. The protein contains a calponin homology domain at its carboxy-terminus, thus it was originally termed the calponin homology-associated smooth muscle protein (CHASM). Experiments completed *in situ *with isolated smooth muscle tissues suggest a physiological role for SMTNL1 in promoting the relaxant actions of PKA/PKG [1,2], and studies with *Smtnl1 *genetic knock-out mice link SMTNL1 with adaptive responses to exercise in both smooth and skeletal muscle [3]. More recent studies have provided indications that SMTNL1 also governs smooth and skeletal muscle adaptations during sexual development and pregnancy [4]. Although less well studied at the molecular level, current data suggests SMTNL1 plays an important regulatory role in smooth muscle contraction and development. The protein is known to interact with tropomyosin [5] and with apo-calmodulin in a Ca^2+ ^dependent manner [6], to inhibit myosin phosphatase activity [2,3] and to regulate the expression of the myosin phosphatase targeting subunit (MYPT1) [4].

As its name suggests, SMTNL1 is closely related to the smoothelin family of proteins (SMTN) that are used as markers for contractile smooth muscle cell differentiation [7,8]. The specific biological role of the two SMTN isoforms, A (short isoform, predominantly visceral expression) and B (long isoform, predominantly vascular expression), remains poorly defined; however, the analysis of mice lacking SMTN-A or -B has revealed critical roles for each of the proteins in intestinal and vascular smooth muscle performance, respectively [9,10]. Interestingly, the SMTN-A and SMTN-B isoforms are expressed from alternative promoters, with the intragenic promoter of SMTN-A residing within exon 10 of the *Smtn *gene [11]. Thus, one *Smtn *gene gives rise to both SMTN-A and SMTN-B mRNA with lengths of 1700 and 3000 nt, respectively. The *Smtn *promoter is controlled by serum response factor (SRF) and myocardin [12]. SRF and myocardin play critical roles in the expression and regulation of growth-responsive genes as well as the expression of virtually all smooth muscle specific genes, such as calponin, myosin heavy chain, α-actin and SM-22 [13,14]. Previous analyses of various smooth muscle and general tissues by immunohistochemistry and Western blotting have revealed strong expression of SMTNL1 in MHC-2A skeletal muscle fibers, moderate expression in smooth muscle tissues (e.g., bladder, ileum, uterus and aorta) and no expression in MHC-I/b cardiac muscle [3]. Given that SMTNL1 is expressed in multiple muscle types, it was expected that *Smtnl1 *transcriptional regulation might differ from the other smoothelin family members.

To date, the contribution of the SMTNL1 protein in smooth muscle contraction has been examined *in vitro *and *in vivo *[1-6], but investigations of its gene and transcriptional regulation are still lacking. Thus, in this study, we identify and characterize key regulatory elements of the promoter region in the mouse *Smtnl1 *gene. This region contains a TSS mapped to a location 119 bp downstream of the NCBI-predicted site. Our data demonstrate that a proximal promoter region is located within 118 bp of the TSS site and provide molecular insights into the regulation of the *Smtnl1 *gene.

## Results and Discussion

### PCR analysis of Smtnl1 transcript

**Figure 1:**
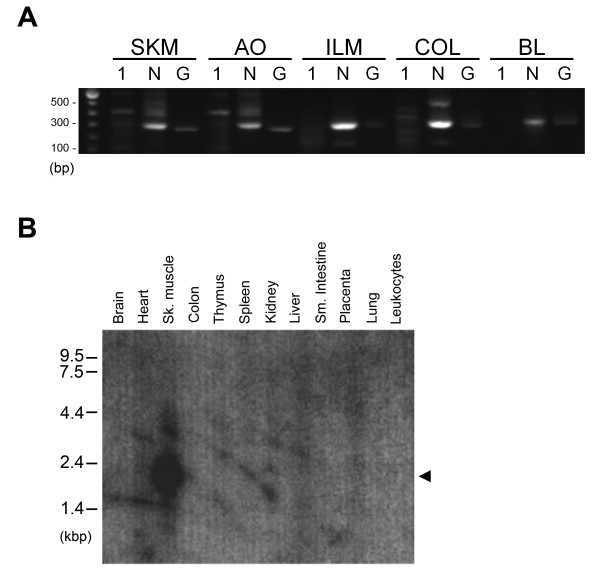
**Expression profile of *Smtnl1 *in various mouse and human tissues**. In **(A)**, an RT-PCR reaction (denoted **1**; expected size, 440 bp) was performed on total RNA extracted from mouse skeletal muscle (SKM) and smooth muscle dissected from aorta (AO), ileum (ILM), colon (COL) and urinary bladder (BL). The RT-PCR was followed by nested PCR (denoted **N**; expected size, 313 bp) to increase the specificity and sensitivity of the detection. RT-PCR reactions of GAPDH (denoted **G**; expected size, 240 bp) was performed to control for the integrity of the extracted mRNA. Results are representative of replicate PCR reactions completed on cDNA synthesized from two separate mice. In **(B)**, Northern blot analysis of *Smtnl1 *expression in human tissues was completed on a Clontech multi-tissue membrane with 1 μg polyA+ RNA loaded per lane. The membrane was hybridized overnight with a ^32^P-labeled *Smtnl1 *cDNA probe (1 - 1038 bp) and exposed to X-ray film. The major human *Smtnl1 *transcript of 2.1-kb is indicated by the arrowhead.

We first examined the pattern of *Smtnl1 *expression in skeletal muscle and representative smooth muscle tissues with RT-PCR. As shown in Figure 1A, the *Smtnl1 *transcript is expressed at very low levels. *Smtnl1 *expression in skeletal and aortic smooth muscle tissues was detectable following 35-cycles of amplification; however, transcript was not detected by RT-PCR in other smooth muscle beds. To increase the sensitivity of detection, we then performed nested PCR reactions on the primary products. This step also increased the specificity of the PCR reaction and permitted qualitative verification of *Smtnl1 *expression. We detected *Smtnl1 *expression by nested PCR in the muscle tissues selected for analysis. The nested-PCR amplicons were sequenced and align 100% to the *Smtnl1 *sequence. Due to its low abundance, we were unable to accurately assess the expression levels in different tissues with qPCR techniques.

We also investigated the expression of mRNA encoding SMTNL1 transcripts in various human tissues with Northern blot analysis. For this study, we used a 1.0-kb 5'-probe based on mouse *Smtnl1 *and moderate stringency washes. The probe was generated to include the sequence encoding the N-terminal 273 amino acid residues of SMTNL1 in order to avoid cross-reactivity with other transcripts that possess sequence encoding a calponin homology (CH)-domain, such as the smoothelins. A single transcript of ~2.1-kb was detected in skeletal muscle (Figure 1B) and correlated with the predicted mRNA size of 1.8-kb. No other mRNA species were identified, implying that a single *Smtnl1 *transcript exists in skeletal muscle. Although we obtained a strong signal for *Smtnl1 *mRNA in skeletal muscle, northern signals were not detected in tissues with high vascular content such as brain and kidney or in tissues with high smooth muscle content (e.g., placenta, small intestine or colon). As seen from our nested-PCR results, a number of smooth muscle tissues do possess *Smtnl1 *mRNA, but at much lower levels than skeletal muscle which might account for the inability to detect expression by Northern blotting. These data agree with protein distributions as determined previously by Western blotting [3]. Interestingly, recent studies have revealed that exercise- and pregnancy-induced alterations in SMTNL1 expression were linked to functional adaptations in skeletal and smooth muscle contractile performances. Given these previous findings, it will be important to generate an accurate tool to quantitatively analyze *Smtnl1 *transcript levels in human tissues and/or mouse models of disease.

### Smtnl1 transcription start site mapping

The murine *Smtnl1 *gene [GenBank: 68678] is located on chromosome 2, E1 and spans 11.48-kb with 8 exons. The mRNA sequence [GenBank: NM_024230] has been annotated using bioinformatic and curatorial analyses in the NCBI Refseq database. A thymidine residue at -162 bp from the translational start site (AUG) within the *Smtnl1 *mRNA was assigned as the provisional transcriptional start site (TSS). This positions the ATG at the start of exon 2 while exon 1 (162 nt) is not translated. To verify the location of the TSS in the *Smtnl1 *gene, we performed 5'-RACE experiments on murine smooth and skeletal total RNA. Three reverse, gene-specific primers were designed for the PCR step that follows cDNA synthesis in the 5'-RACE process (see Table 1). Primer GSP3 and GSP 2 anneal just downstream of the TSS and upstream of the AUG (expected PCR product 63 bp and 176 bp, respectively). Primer GSP1 anneals just upstream and outside of the CH domain in exon 8 (expected PCR product 1479 bp).

**Table 1:** PCR primers used in the analysis of *Smtnl1*.

RT-PCR & nested PCR	
mSmtnl1-F1	AAGAAGGATCGAGCACCAGAAC
mSmtnl1-R1	ACACACTTGGAATCGGGCAC
mSmtnl1-nested-F1	GGAAGGCCATCATGGACAAATTT
mSmtnl1-nested-R1	GCACAGTCGGCTAGTTTCTCTG

	
**5'-RACE**	

5'-CDS	dT_25_VN (N = A, C, G, or T; V = A, G, or C)
SMART II A	AAGCAGTGGTATCAACGCAGAGTACGCGGG
UPM	5:1 mixture of (1): (2) (1) CTAATACGACTCACTATAGCAAGCAGTGGTATCAACGCAGAGT (2) CTAATACGACTCACTATAGC
NUPM	AAGCAGTGGTATCAACGCAGAGT
GSP1	CCACCTCCAGCAGCTGGGCACAGTCGGCTAG
GSP2	GCCTCGTTCTAGCCGAGCTGCCTCACTCTCCAT
GSP3	CACCCCACCTTCAAGAGGCCCGGGCTTCG
NGSP1	CACCCCACCTTCAAGAGGC
NGSP2	CCAACTCCTTTGTGTCACTTCCG

	
**RPA**	

RPA2	TAATACGACTCACTATAGGGCTGATCAGATGCTAGGTACACTT GCTTCCACCACCCCTTGCTTCCACC
T7 promoter	TAATACGACTCACTATAGGG
RPA-NCS	GAGAGGGACGCAGCAGCGTG
RPA-NCA	GGTTCTGCCTGCAGCTCGG
RPA-M1-2S	TAGAACGAGGCAAGAGCTCC
RPA-M1-2A	GTTCTGCCCAATCTGAAGGC
RPA-FR1	GCCACCTGTCAGATCTTCGG
RPA-RT7	TAATACGACTCACTATAGGGCTGATCAGATGCTAGGTACAG TTCTGCCCAATCTGAAG

	
**Promoter Truncations**	

+100 bp LUC R1	GTGGGATCCGTGAAAGGTAAGAATAGG
0 bp LUC F1	CTAGCCTTCTAGAACGAGGCAAGAGCTCC
-118 bp LUC F2	CTAGAATTCCTTCCCGAAGCCCGGGC
-218 bp LUC F3	CTCGAATTCGCAGCGTGATTGAAAGATG
-468 bp LUC F4	CTAGAATTCCCCCTCAGCAGCTACTTTG
-767 bp LUC F5	CTCGAATTCCGTTGGGAACTGGGAATTCCG
-1350 bp LUC F6	CTAGAATTCGACAAAGCTTGGGGTCTATATC
-1637 bp LUC F7	CTAGAATTCCTCTTAACCTCCAAGACATC
-1869 bp LUC F8	CTAGAATTCCTCTTAACCTCCAAGACATC
-2368 bp LUC F9	CTAGAATTCCAAAGTCCTATTGCTTGCACC
-2759 bp LUC F10	GCTGAATTCGAATTATCTCATGGACTTGCCTAGAG

**Figure 2:**
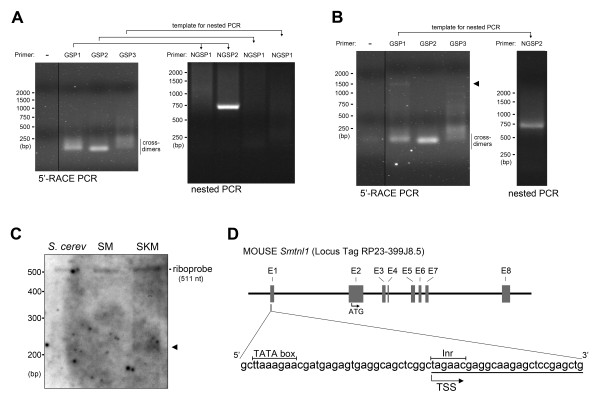
**Identification of the transcription start site of *Smtnl1 *by rapid amplification of cDNA 5'-ends (5'-RACE) and ribonuclease protection assay (RPA)**. 5'-RACE reactions were performed on cDNA synthesized from smooth **(A) **or skeletal **(B) **muscle mRNA with primers specific for *Smtnl1*. Representative ethidium bromide-stained agarose gels of PCR products are shown. A 5'-RACE CDS primer (Stratagene) and three *Smtnl1 *gene specific primers (GSPs) were used with expected product sizes: GSP1 (1479 bp), GSP2 (176 bp) and GSP3 (83 bp). The 5'-RACE product from each reaction was used as template in a subsequent nested PCR reaction. The expected sizes of the nested PCR products were: NGSP1 (63 bp) and NGSP2 (823 bp). The arrowhead in **(B) **indicates the PCR product visible during the primary amplification when skeletal muscle cDNA was used as template for 5'-RACE with GSP1. The DNA ladder markers are indicated on the *left*. In **(C)**, ribonuclease protection assay (RPA) analysis was performed on 10 μg of total RNA from smooth (SM) and skeletal (SKM) muscle. A biotin-labeled *Smtnl1 *riboprobe was generated to span -112 bp to the TSS, joined to exon 1 and exon 2 from the *Smtnl1 *mRNA, up to + 319 bp. Total RNA from *S. cerevisiae *(10 μg) was used instead of muscle RNA as a negative control. The band appearing with skeletal muscle mRNA is marked by an arrow. The DNA ladder markers are indicated on the *left*. In **(D)**, the promoter region, eight exons (E1-E8) and 7 intronic DNA sections are shown for the mouse *Smtnl1 *gene. The positions of the start codon (ATG), transcriptional start site (TSS) defined by 5'-RACE, the TATA box and initiator sequence (Inr) are also indicated. The nucleotides identified by sequencing the cDNA clones (n = 5) obtained from 5'-RACE are underlined.

The 5'-RACE PCR on smooth muscle (Figure 2A) and skeletal muscle (Figure 2B) total RNA produced low (< 250 bp) molecular weight cross-dimer products between the GSP and 5'-RACE UPM primers (as verified by sequencing). When skeletal muscle mRNA was used as template together with primer GSP1, a faint band was visible at approximately 1.5-kb that was close to the expected product size (Figure 2B). To increase specificity and sensitivity, a second round of PCR was performed using nested primers on the products from the 5'-RACE PCR. The expected amplicon sizes with the NCBI-predicted TSS were 63 bp for NGSP1 and 823 bp for NGSP2. A strong band of approximately 700 bp was observed for nested PCR reactions completed on both smooth and skeletal muscle GSP1 PCR products (Figure 2A &2B), somewhat smaller than the expected 823 bp product for the NGSP2 primer. Sequence analysis revealed a partial alignment of the 823 bp NGSP2 product with *Smtnl1*, starting within exon 1.

The 5' end of the product was 119 bp shorter than expected from the NCBI-predicted TSS. To further validate the TSS identified with 5'-RACE, ribonuclease protection assays (RPA) were performed. A ssRNA riboprobe (511 nt) was generated to span both the NCBI-predicted and 5'-RACE-derived TSS. A protected fragment of 369 bp was expected for the NCBI-predicted TSS whereas a 231 bp fragment was expected for the newly derived TSS. As shown in Figure 2C, RPA was completed with smooth and skeletal muscle mRNA and biotin-18-UTP-labeled riboprobe. As expected, the full-length probe was detected near the 500 bp marker. A protected fragment slightly larger than 200 bp was detected in the RPA with the addition of skeletal muscle mRNA. This result further confirms the TSS location to be different from that annotated in the NCBI database, occurring less than 50 nt upstream of the translational start codon. No protected fragment was detected when yeast RNA (control) or smooth muscle RNA was used in the RPA. The latter result was likely due to lack of sensitivity of the RPA conditions for low abundance mRNAs.

Analysis of the sequence located upstream of the start codon (ATG) in murine *Smtnl1 *indicated the presence of an initiator (Inr) sequence and a TATA box (Figure 2D). Therefore, our experimentally mapped *Smtnl1 *TSS corresponded to a thymidine within the Inr sequence (CTAGAAC, where the T was the first transcribed nucleotide) and was located 44 nt upstream of the translational start codon. The putative TATA box sequence was identified at -23 to -31 nt upstream of the new TSS. Further sequence analysis showed that the Inr and TATA sequences of *Smtnl1 *are conserved within a variety of mammalian *Smtnl1 *genes.

The expression profile of *Smtnl1 *differs from the other smoothelin (Smtn) family members that are exclusively expressed in differentiated smooth muscle cells. The *Smtnl1 *promoter was expected to be different since the smoothelins are not expressed in any tissue but smooth muscle cells. Indeed, the promoter sequences of smoothelins and *Smtnl1 *do not bear any resemblance to each other, despite some similarities in the protein-coding region (mainly within the C-terminal calponin homology domain). The SMTN-A and SMTN-B isoforms of the *Smtn *gene are generated by alternative transcriptional start sites [11] that provide selective expression of each isoform in visceral and vascular smooth muscle beds, respectively. Moreover, alternative splicing produces three different C-terminal variants of each SMTN isoform [15]. It is unlikely that multiple SMTNL1 isoforms exist since a single *Smtnl1 *TSS was identified from both skeletal and intestinal smooth muscle total RNA with sequencing of several 5'-RACE clones. All sequences commenced at a thymine located 44 nt upstream of the translational start site (ATG). However, we have not yet completed 5'-RACE on *Smtnl1 *transcripts isolated from vascular tissue. Furthermore, we cannot exclude the presence of multiple C-terminal splice variants since we did not complete 3'-RACE analysis.

### SMTNL1 promoter reporter assay

**Figure 3:**
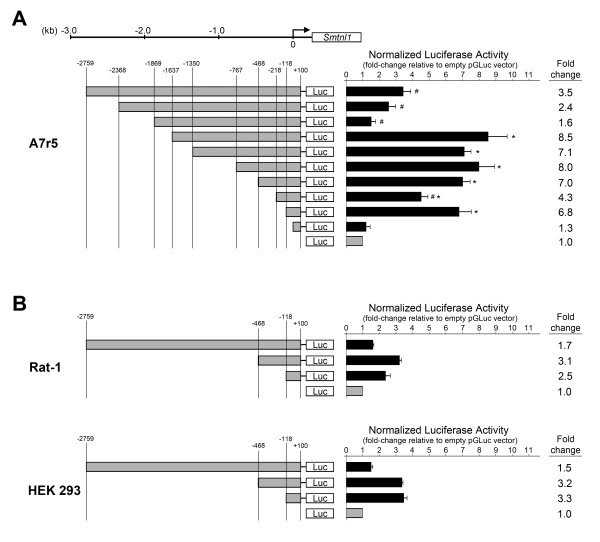
**Identification of enhancer and repressor regions within the *Smtnl1 *promoter**. In **(A)**, various deletions (*left*) of the *Smtnl1 *promoter region spanning +100 bp to -2759 bp were used to drive expression of the *Gaussia *luciferase gene. The positions were numbered from the transcriptional start site (TSS) identified by 5'-RACE in this study. A7r5 cells were transiently co-transfected with the pGLuc reporter and the constitutively active β-galactosidase (β-gal) reporter vector, pbAct-β-gal for normalization. After 48 hr, the media and cell lysates were assayed for luciferase and β-gal activities, respectively. Luciferase activity was normalised to β-gal activity and expressed as fold-increase (*right*) relative to the activity of the empty pGLuc vector. All values are mean ± S.E.M. (n = 5-8). *- significantly different from +100 - 0 bp promoter region; #- significantly different from 0 to -118 bp promoter region. Statistical analysis was completed with ANOVA and Student-Neumann-Keuls *post-hoc *analysis, p < 0.05. Additional studies **(B) **also examined luciferase expression in Rat 1 fibroblast and human embryonic kidney (HEK 293) cell lines 48 hr after transient co-transfection with the pGLuc reporter vector and pbAct-β-gal for normalization. All values are mean ± S.E.M. (n = 3).

To locate *cis*-elements in the core promoter region that modulate expression of *Smtnl1*, we constructed deletion mutants of the *Smtnl1 *upstream sequence and monitored *Gaussia *luciferase reporter activities in rat aortic A7r5 smooth muscle cells. Promoter sequences were generated spanning up to 2.7-kb upstream of the newly derived *Smtnl1 *TSS and cloned into a promoterless pGLuc luciferase vector. Figure 3A shows the relative luciferase activity using progressively shorter promoter constructs. A 3.5-fold induction was observed for the upstream region spanning +100 to -2759. Deletion of the region from -1637 to -1869 increased the luciferase induction to 8.5-fold while removal of the sequence from -468 to -1637 elicited no further effect. The minimal sequence establishing the proximal promoter (0 to -118) provided a 7-fold luciferase induction. Finally, the first 100 bases downstream of the new TSS had no activity. These data reveal two regions of promoter sequence that functioned as enhancer elements, -218 to -468 and 0 to -118. Furthermore, two inhibitory elements were also defined, -1637 to -1869 and -118 to -218. These results demonstrate that the first 118 bp upstream of the *Smtnl1 *TSS could significantly enhance luciferase production, suggesting that the core promoter was located in this region. Importantly, the region of the core promoter did not include the TSS previously annotated in NCBI. Our data confirms that the region immediately upstream of the NCBI-annotated TSS could not enhance expression, which further verifies our newly identified TSS downstream of the NCBI-annotated TSS. We further investigated the sequence of the first 118 bp upstream of the TSS for possible promoter elements. A TTAAA motif, 31 nt upstream of the TSS, was 100% conserved amongst all the mammalian *Smtnl1 *promoters investigated and is predicted to serve as a TATA-box. We were unable to identify a CAAT element.

The promoter region just upstream and including the TSS is highly conserved in mammals, emphasizing its importance. The TSS of some mammals is not annotated in this region, and this might be due to difficulties in identifying 5'-UTRs within the corresponding ESTs. Further corroborating this possibility was the distance between the TSS (equivalent to murine exon 1) and the translational start-site (ATG) in murine exon 2. In all mammals, this distance was similarly expansive (e.g., 3400 bp (dog) to 5200 bp (cow)). We propose that all mammals investigated have a TSS located downstream of the conserved region, similar to the murine promoter. This would add an additional 5'-UTR exon 1 to human, rat and horse *Smtnl1*.

We also investigated whether the pattern of luciferase inductions observed with the *Smtnl1 *promoter sequences resulted from a specific set of transcription factors unique to A7r5 smooth muscle cells. For this, the activities of three *Smtnl1 *promoter constructs were also assessed in HEK 293 (human embryonic kidney) and Rat-1 (rat fibroblast) cell lines (Figure 3B). The same pattern of luciferase induction was seen in these cell lines; however, the maximal activation measured was only 3-fold, a significant reduction when compared to the maximal activity found in A7r5 smooth muscle cells. The identified *cis*-acting promoter spanned at least 1869 bp and could up- or down-regulate expression in A7r5, Rat-1 and HEK 293 cells, depending on which region was present. However, the much higher maximum activation in smooth muscle cells compared to fibroblast and kidney cells suggests restrictive control of *Smtnl1 *expression in non-muscle cells and emphasizes the importance of SMTNL1 expression in muscle contractile function.

### In silico analysis of putative transcription factor binding sites within Smtnl1

**Figure 4:**
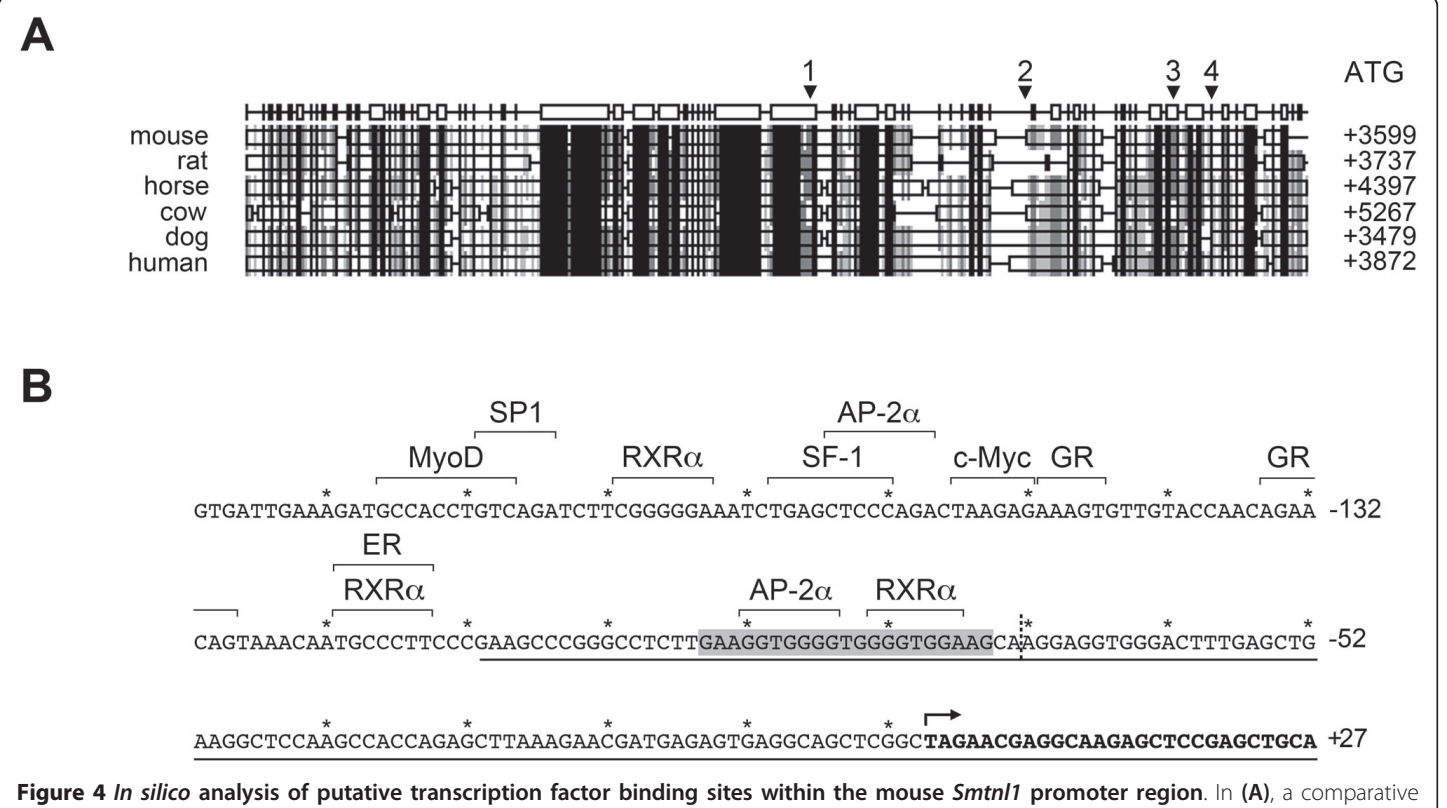
***In silico *analysis of putative transcription factor binding sites within the mouse *Smtnl1 *promoter region**. In **(A)**, a comparative analysis of mammalian *Smtnl1 *promoter regions was completed. The promoter sequences were aligned using CLUSTALW and Genedoc. The sequence similarities are: black, 100%; dark grey, 99 - 80%; light grey, 80 - 60%; and white, 60 - 0% conservation. The NCBI-predicted TSS of mouse (1), dog (2) and cow (3) *Smtnl1 *genes as well as the newly defined murine TSS (4) and distance from the promoter to the start codon (ATG) are indicated. The highly conserved region was further investigated in **(B)**. The *Smtnl1 *sequence located immediately upstream of the newly defined TSS (indicated by bold arrow; downstream sequence in bold) was investigated for putative transcription factor binding sites as indicated. The grey box outlines the mirror repeat sequence (MRS). The dashed line shows the NCBI-derived TSS. The underlined region highlights the *Smtnl1 *promoter sequence that strongly enhanced luciferase reporter activity, and "*" indicate 10 nucleotide sections of sequence.

A comparison of TSS sites in various mammalian *Smtnl1 *genes was undertaken, and BLAST searches revealed that exon 1 is not translated and the murine ATG start codon is located at the beginning of exon 2. In the case of some mammals (i.e., human, rat and horse), the *Smtnl1 *gene starts with the ATG in exon 1 and is analogous to murine exon 2. Further investigation of these *Smtnl1 *variants revealed the location of a genomic region that has not been annotated. This region lays several thousand bases upstream with high similarity to the murine exon 1. Comparison of various mammalian SMTNL1 protein sequences revealed the highest conservation at the N- and C-termini (aa 1 - 17 as well as aa 209 - 459). Thus, it is not surprising that, based on protein sequence and gene sequence alignment, the exon including the start codon was annotated in all mammals while the upstream exon 1 was sometimes not annotated. In agreement with the observation that exon 1 is often located far upstream of exon 2, there is approximately 3000 to 4000 bp separating the conserved region corresponding to the mouse 5'-UTR and the ATG start codon of *Smtnl1 *from various mammalian species (Figure 4A). Based on these findings, it is reasonable to predict that the human *Smtnl1 *TSS lies upstream of the region homolog to murine *smtnl1 *exon 1, even though it remains to be annotated. Indeed, the region just upstream of the murine TSS is highly conserved in mammals, with > 75% percent identity (Figure 4A).

**Figure 5:**
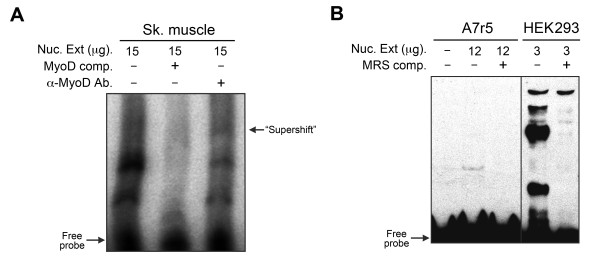
**EMSAs of the putative MyoD-binding and mirror repeat sequence in the murine *Smtnl1 *promoter**. [γ-^32^P]-labeled oligonucleotide probes (3 pmol) were incubated with nuclear extracts isolated from murine skeletal muscle (for MyoD, in **A**) or A7r5 and HEK293 cells (for mirror repeat sequence (MRS), in **B**). In some experiments, 200-fold excess unlabeled competitors were added prior to incubation with ^32^P-labeled probes. For supershift analysis, anti-MyoD antibody (1 μg) was introduced with nuclear extracts following the addition of labeled probe. Results are representative of n = 3 independent experiments.

The highest conservation in the murine *Smtnl1 *5'-UTR sequence was located between -80 bp and -220 bp relative to the newly derived TSS, suggesting functional importance. Indeed, the region corresponds in part to the strongly activating construct determined with the luciferase reporter assay (i.e., +100 to -118). We searched for transcription factor binding sites (TFBS) in this region using the PATCH program that utilizes the TRANSFAC^® ^database of *cis*-acting transcription factors [16,17]. Possible mammalian TFBSs in this region include AP2α, SF-1, RXRα, SP1, ER and c-myc amongst others (Figure 4B). Intriguingly, this region of the *Smtnl1 *promoter also possesses a putative TFBS for MyoD (myogenic differentiation-1), a transcriptional activator of muscle-specific genes [18], located 190 bp upstream of the TSS. MyoD shows highest transcriptional activation when dimerized with another MyoD or other family members (e.g., Sp1, Mef2 and Pbx) [19,20]. Indeed, possible non-canonical sites for MyoD as well as Sp1 are located adjacent to the primary MyoD site in the *Smtnl1 *promoter. Thus, regulation of *Smtnl1 *expression via MyoD would explain the robust expression found in skeletal muscle when compared to other tissues. We completed EMSAs with nuclear extracts from mouse skeletal muscle and DNA probes derived from the MyoD site in the *Smtnl1 *promoter (Figure 5A). Gel shifts were suggestive of protein binding to the *Smtnl1 *MyoD sequence and could be inhibited with addition of excess unlabelled probe as a competitive inhibitor. Moreover, EMSAs completed in the presence of MyoD antibody exhibited a defined "supershifting" band.

The highest density of putative TFBSs lay within the sequence GAAGGTGGGGTGGGGTGGAAG of *Smtnl1 *that forms a 20 bp mirror-repeat sequence (MRS). Mirror repeats are commonly associated with the formation of an intramolecular triple-helical conformation termed H-DNA [21,22]. Many promoters, especially those integral to genes involved in growth regulation, have H-DNA forming mirror repeats. The *Smtnl1 *MRS lies within a region of high transcriptional activation as determined by luciferase reporter assay, and it is possible that this region is of paramount importance for *Smtnl1 *expression. EMSAs completed with MRS oligonucleotide probes and nuclear extracts from A7r5 cells demonstrated a single intense band (Figure 5B). This binding pattern was significantly altered when nuclear extracts from HEK293 cells were used; in this case, multiple bands were observed. The results are suggestive of transcription factor binding to the MRS; however, more in depth investigation will be required to identify the specific proteins involved.

Other transcription factors are of interest based on their function and our knowledge of SMTNL1. Putative binding sites for the nuclear hormone receptor steroidogenic factor-1 (SF-1) as well as estrogen receptor (ER) are present. These receptors might be responsible for the observed differences in *Smtnl1 *expression during sexual development in males [3] as well as during pregnancy in females [4]. RXRα (retinoid × receptor alpha) modulates the actions of other hormone receptors and plays a role in myogenesis. RXRα is also responsible for the activation of MyoD and deficiency in RXRα causes defects in skeletal muscle development [23]. Furthermore, RXRα is a binding partner for PPAR (peroxisome proliferator-activated receptor) transcription factors [24]. In turn, PPARγ regulates endurance-exercise induced fiber type remodeling [25]. Since endurance-exercise caused up-regulation of *Smtnl1*, RXRα together with PPARγ might be the responsible factors. Notable is the absence of a consensus CC-A/T rich-GG (CArG) box, the binding site for serum response factor (SRF) that is present in smooth muscle restricted genes [26,27]. This distinguishes *Smtnl1 *from the related *Smtn *genes and is further manifested by the expression of Smtnl1 in skeletal muscle [3]. Of the numerous transcription factors predicted to bind to the proximal promoter, several could be responsible for the adaptive expression of *Smtnl1 *after endurance-exercise, pregnancy and sexual development in smooth and skeletal muscle. This will be subject to further research.

## Conclusions

In the present study, we identified *Smtnl1 *expression in all types of smooth muscle tissue (e.g., phasic and tonic; vascular and visceral) examined, albeit at lower levels than found in skeletal muscle. Current evidence supports a key role for SMTNL1 in adaptive responses to physiological stresses (e.g., pregnancy, exercise and sepsis). Therefore, defining key elements of the murine *Smtnl1 *gene will enable better understanding of mechanisms for smooth and skeletal muscle contractile plasticity in response to physiological stresses.

## Methods

### RNA samples and cell culture

Mouse total skeletal muscle RNA and mouse total smooth muscle RNA (from small intestine) were purchased from Clontech Laboratories (Mountain View, CA). Rat aortic smooth muscle (A7r5, [ATCC:CRL-1444]), human embryonic kidney (HEK 293, [ATCC:CRL-1573]) and rodent fibroblast (Rat 1, [ATCC:CRL-2210]) cells were maintained in Dulbecco's Modified Eagle Medium (DMEM) supplemented with 10% fetal bovine serum (FBS) in a humidified tissue culture incubator at 37°C with 5% CO_2_. DMEM and FBS were purchased from Invitrogen (Carlsbad, CA).

### Reverse transcription Polymerase Chain Reaction (RT-PCR)

For all RT-PCR reactions, the 'standard PCR' kit from Fermentas (Glen Burnie, MD) was used. All reactions were carried out using a thermal cycler (Eppendorf Mastercycler; Westbury, NY). Skeletal muscle as well as aorta, ileum and colonic smooth muscle tissues were removed from male mice (C57Bl/6) that had been anesthesized and euthanized according to protocols approved by the University of Calgary Animal Care and Use Committee. Total RNA and mRNA were extracted using the RNeasy Mini kit (Qiagen) or Ambion Micro Poly(A) Pure extraction kit, respectively. First strand cDNA was synthesized using the Sensiscript RT kit (Qiagen). For the PCR amplification, Phusion Taq DNA polymerase (New England Biolabs) was used with the gene-specific primers mSmtnl1-F1, mSmtnl1-R1, mSmtnl1-nested-F1 and mSmtnl1-nested-R1. The primer sequences are noted in Supplementary Table 1. Primers were selected to be unique to *Smtnl1 *mRNA and did not occur within the transcripts of the other smoothelin family members. The nested-RT-PCR reactions were used because they offer increased specificity and sensitivity. RT-PCR reactions for GAPDH were performed on all RNA samples to verify the integrity of the RNA.

### Northern Blot

Mouse *Smtnl1 *cDNA (1 - 1038 bp) was radiolabeled with [α^32^P]-dCTP by the random-priming method using a PrimeIt II Random Primer Labeling kit (Stratagene). Unincorporated nucleotide was removed with a NucAway Spin Column (Ambion). A multiple tissue Northern blot that contained poly(A)+ RNA from a variety of human tissues was purchased from Clontech. The poly(A)+ RNA membrane was prehybridized in ExpressHyb solution for 30 min at 68°C. Radiolabeled cDNA probe (~5 × 10^6 ^cpm/mL) was denatured at 100°C for 2 min then rapidly chilled on ice. The membrane was incubated with the radiolabeled probe in 5 mL of fresh ExpressHyb with continuous shaking for 1 h at 68°C. Following hybridization, the blot was washed for 30 min at 23°C with 2× SSC (300 mM NaCl, 30 mM Na citrate, pH 7.0), 0.5% SDS (several washes) and twice for 20 min with 0.1× SSC (15 mM NaCl, 1.5 mM Na citrate, pH 7.0), 0.1% SDS at 50°C. The membrane was then subjected to autoradiography.

### Rapid Amplification of 5' cDNA ends (5'-RACE)

The SMART RACE cDNA Amplification kit from Clontech Laboratories was used to amplify 5'-cDNA ends of mouse smooth and skeletal muscle mRNA. First strand cDNA synthesis was carried out according to the manufacturer's instructions with mouse total RNAs (Clonetech, 1 μg) from intestinal smooth muscle or skeletal muscle, 5' CDS primer A, SMART II A oligonucleotide and TITANIUM *Taq *DNA polymerase (Advantage 2 PCR System, Clontech). The 5'-RACE PCR reaction was performed according to the manufacturer's protocol using Universal Primer A mix (UPM, forward primer) and one of three *Smtnl1 *gene specific primers (GSP1-3; reverse primers). The procedure incorporates an additional 50 bp to the 5'-end of the PCR product. To verify the RACE products, nested 5'-RACE PCR was carried out using nested UPM (NUPM) forward primer and one of three nested *Smtnl1 *gene specific reverse primers (NGSP1, NGSP2). The nested RACE products were separated on a 1% agarose gel, extracted and cloned into the TOPO-TA vector (Invitrogen, Carlsbad, CA). All nested RACE products were sequenced at the University of Calgary DNA core service.

### Ribonuclease protection assay (RPA)

To verify the translation start site (TSS), RPA was performed on murine smooth and skeletal muscle mRNA. The template for the riboprobe was a chimera of murine genomic DNA, comprising the 5'-UTR and exon 1 of the *Smtnl1 *gene (-52 nt to + 160 nt relative to the NCBI-predicted TSS [Gene ID: 68678]) plus the 5'-end of exon 2 taken from *Smtnl1 *(+161 nt to +319 nt [GenBank: NM_024230.1]) and a T7 primer overhang. The template was generated using standard PCR methods: the BAC clone RP23-125A8 (Children's Hospital Oakland Research Institute, Oakland, CA) and primers RPA-NCS and RPA-NCA were used for the genomic portion, and the product of the 5'-RACE together with primers RPA-M1-2 S and RPA-M1-2A was used as the mRNA part. A second PCR with primers RPA-FR1 and RPA-RT7 engineered the T7 promoter into the RPA-template. The riboprobe was synthesized using T7 DNA dependent RNA transcriptase (New England Biolabs, Ipswich, MA) according to the manufacturer's instructions. To label the riboprobe, biotin-18-UTP/UTP (1:5 ratio) was used in the reaction mix (Invitrogen). For the RPA, the RPA III kit (Ambion, Austin, TX) was used with 10 μg mRNA and 100 pg riboprobe. Briefly, murine mRNA and the riboprobe were hybridized to form dsRNA (40.5°C, overnight). Then, any remaining single-stranded mRNA and riboprobe were digested with RNase. The protected RNA was resolved by denaturing PAGE and transferred to a SensiBlot nylon membrane (Fermentas, Glen Burnie, MD). The remaining biotin-18-UTP-labeled riboprobe was detected using Ambion's Brightstar Biodetect kit according to the manufacturer's instructions.

### Luciferase Assay

A luciferase reporter assay was used to investigate the putative *Smtnl1 *promoter sites in the 5'-flanking region. The *Smtnl1 *gene and 5'-flanking sequence spanning bp 84662089 to bp 84651332 of mouse chromosome 2 was obtained from BAC clone RP23-125A8. Different lengths of *Smtnl1 *5'-flanking DNA were generated by PCR with the BAC plasmid as template and cloned into the pGLuc-Basic vector (New England Biolabs, Ipswich, MA). The antisense primer utilized for all constructs engineered a BamHI site at +100 bp downstream of the transcription start site identified by our previous 5'-RACE/RPA analysis. Ten different sense primers were used, adding an EcoRI site for cloning into the pGLuc-Basic vector. All clones were verified by DNA sequencing. Using Metafectene-Pro (Biontex Laboratories, München, Germany) and following the manufacturer's protocols, we transiently transfected A7r5, HEK 293 or Rat-1 cells with pGLuc-Basic vectors (5 μg) containing the various *Smtnl1 *promoter constructs and pbActb-gal vector (1 μg). The pbActb-gal vector expresses β-galactosidase under the constitutively active β-actin promoter and was a kind gift from Dr. J. Cross (University of Calgary). Twenty-four hours before transfection, cells were plated in 30-mm dishes to produce a density of ~80% confluence. At 48 hours post-transfection, the media was assayed for luciferase activity using the Gaussian Luciferase Assay kit (New England Biolabs) and cell lysates were assayed for β-galactosidase activity, in quadruplicate samples of three independent experiments. To account for differences in transfection efficiency, the luciferase activity was normalized to β-galactosidase activity and expressed as % change relative to the activity of empty pGLuc-Basic vector (defined as 100%). As a positive control, pGLuc-CMV was used in separate transfections.

### Electrophoretic mobility shift assay (EMSA)

DNA binding was assessed by EMSAs with standard procedures [28] using nuclear extracts prepared from A7r5 cells or mouse skeletal muscle tissue (hind-limb). Double-stranded oligonucleotide probes containing the mirror-repeat (MRS) and putative MyoD binding sequences located within the *Smtnl1 *promoter region were synthesized (sense strands: MRS: 5'-GAAGGTGGGGTGGGGTGGAAG-3'; MyoD: 5'-ATTGAAAGATGCCACCTGTCAGATCT-3'). Each oligonucleotide was annealed to its complementary strand, end-labeled with [γ-^32^P]-ATP using T4 polynucleotide kinase and then purified with G-25 Sepharose mini-columns. Binding reactions were carried out in a mixture containing 1-15 μg nuclear extract proteins in 20 μL of binding buffer (10 mM Tris, 50 mM KCl, 1 mM DTT, 5% glycerol, pH 7.5). Radiolabeled, double-stranded oligonucleotide probe (3 pmol) was added, and mixtures were incubated at room temperature for 30 min prior to resolution on 5% non-denaturing polyacrylamide gels (1× TBE). The gels were then dried, and the radioactive bands were developed using a Storm phosphoimager (GE Healthcare). For competition experiments, unlabeled MyoD or MRS oligonucleotide competitor (200-fold excess) was added to the reaction mixture before incubation with the radiolabeled probe. For MyoD supershift assays, the reaction mixtures were incubated with 1 μg of anti-MyoD antibody prior to the addition of radiolabeled probe.

### Bioinformatic promoter analysis

The 5'-UTR of the murine *Smtnl1 *was searched against mammalian genomes using the BLAST algorithm. Regions with high alignment score (expectancy value E < 10^-10^) were aligned using CLUSTALX and GeneDoc after visual inspection. All mammalian sequences aligning to the murine gene (i.e., bp 25704307 to 25703966 of the reverse strand of GenBank: NT-039297.7; 197 bp upstream of the TSS defined by NCBI) were selected for further investigation. The sequence identity was approximately 54%. The region between +38 to -94 relative to the NCBI TSS (-79 to -211 of the newly defined TSS) was termed highly conserved with an identity of >72% between all sequences. This region of highly conserved sequence was analyzed with the PATCH program for putative transcription factor binding sites based on the Transfac^® ^database [16,17].

## List of Abbreviations

bp: base pair; cGMP: cyclic-guanidine monophosphate; DMEM: Dulbecco's Modified Eagle Medium; EMSA: electrophoretic mobility shift assay; FBS: fetal bovine serum; MRS: mirror repeat sequence; MyoD: myogenic differentiation-1; nt: nucleotide; PKA: protein kinase A; PKG: protein kinase G; 5'-RACE: rapid amplification of 5' cDNA ends; 3'-RACE: rapid amplification of 3' ends; RPA: ribonuclease protection assay; SMTN: smoothelin; SMTNL1: smoothelin-like 1; SRF: serum response factor; TFBS: transcription factor binding site; TSS: transcriptional start site; 5'-UTR: 5' untranslated region.

## Authors' contributions

AU-L carried out MyoD EMSAs and bioinformatic work, interpreted the data and drafted the manuscript. ST performed RT-PCR for Smtnl1 in mouse tissues. SM carried out 5' RACE, RPA and luciferase assay analyses. RW contributed to the design of 5'-RACE, RPA and nested RT-PCR experiments as well as the interpretation of the data. MB performed the northern blot. JM conceived and designed the study, interpreted the data and revised the manuscript for intellectual content. All authors read and approved the final manuscript.
